# Benefits and Pitfalls of the Perceval Sutureless Bioprosthesis

**DOI:** 10.3389/fcvm.2021.789392

**Published:** 2022-01-05

**Authors:** Aleksander Dokollari, Basel Ramlawi, Gianluca Torregrossa, Michel Pompeu Sá, Serge Sicouri, Edvin Prifti, Sandro Gelsomino, Massimo Bonacchi

**Affiliations:** ^1^St. Michael's Hospital, Toronto, ON, Canada; ^2^Department of Cardiac Surgery, Lankenau Heart Institute, Wynnewood, PA, United States; ^3^Department of Cardiac Surgery Research, Lankenau Institute for Medical Research, Wynnewood, PA, United States; ^4^Mother Teresa Hospital, University of Tirana, Tirana, Albania; ^5^Department of Cardiac Surgery, Maastricht University Medical Center, Maastricht, Netherlands; ^6^Department of Cardiothoracic Surgery, Cardiovascular Research Institute Maastricht—CARIM, Maastricht, Netherlands; ^7^Cardiac Surgery Unit, Department of Experimental and Clinical Medicine, University of Florence, Florence, Italy

**Keywords:** benefits, pitfalls, Perceval, sutureless, review, sutureless valve replacement

## Abstract

**Objective:** To highlight the main target points covered by clinical studies on the Perceval sutureless valve for surgical aortic valve replacement (SAVR) and raise a point of discussion for further expansion of its use when compared with stented bioprostheses (SB) and transcatheter aortic valve replacement (TAVR).

**Methods:** We reviewed clinical trials and retrospective studies published up to date and compared the outcomes in terms of mortality, myocardial infarction (MI) stroke, paravalvular leak (PVL), permanent pacemaker implantation (PPI), bleeding and long-term outcomes.

**Results:** Clinical studies showed that 30-day mortality ranged from 0–4% for Perceval and 2.9–7% for TAVR. The incidence of PVL (Perceval 1.9–19.4 vs. TAVR 9–53.5%), PPI (Perceval 2–11.2 vs. TAVR 4.9–25.5%), stroke (Perceval 0 vs. TAVR 0–2.8%), MI (Perceval 0 vs. TAVR 0–3.5%), were all higher in the TAVR group. Compared to other SB, mortality ranged from 0–6.4% for Perceval and 0–5.9% for SB. The incidence of PVR (Perceval 1–19.4 vs. SB 0–1%), PPI (Perceval 2–10.7 vs. SB 1.8–8.5%), stroke (Perceval 0–3.7 vs. SB 1.8–7.3%) and MI (Perceval 0–7.8 vs. SB 0–4.3%) were comparable among the groups. In patients with a bicuspid aortic valve, mortality rate was (0–4%) and PVL incidence was (0–2.3%). However, there was a high incidence of PPI (0–20%), and stroke (0–8%). Long-term survival ranged between 96.7–98.6%.

**Conclusions:** The Perceval bioprosthesis has proved to be a reliable prosthesis for surgical aortic valve replacement due to its implantation speed, the reduced cardiopulmonary bypass time, the reduced aortic cross-clamp time and the shorter intensive care unit and hospital length of stay.

## Introduction

Surgical aortic valve replacement (SAVR) with the sutureless self-expanding Perceval aortic bioprosthesis (LivaNova Group, Milan, Italy) was developed to combine the advantages of the transcatheter aortic valve replacement (TAVR) procedure, allowing for a fast implantation with no need for suturing, with the benefits of a conventional surgical approach owing to the possibility of removing the native valve along with the calcifications. The valve has grown in popularity mostly due to the reduced cardiopulmonary bypass (CPB) time (1), the improved myocardial recovery time and its application in minimally invasive cardiac surgery (MICS) procedures (2). In addition, the three PARTNER clinical trials' (3–5), the SURTAVI trial (6) and other observational cohort studies (7, 8) have evidenced the non-inferiority of TAVR vs. SAVR. In this context, some reports of successful valve-in-valve TAVR in bioprostheses with structural valve deterioration (SVD) have generated enthusiasm particularly for future applications (9, 10). In addition, other outcomes of the valve include improved hemodynamics, a self-expanding radial force, usage in hostile roots, enhanced surgical and recovery speed, and enabling minimally invasive cardiac surgery procedures. However, many points deserve to be highlighted such as the impact of permanent pacemaker implantation (PPI) after SAVR, the application of the sutureless bioprostheses in patients with bicuspid aortic valves (BAV), the impact of thrombocytopenia on the survival rate and the implantation of this bioprostheses in patients with small aortic annuli.

The goal of this review is to highlight the main target points covered by clinical studies and raise a point of discussion for further expansion of the use of Perceval.

## Materials and Methods

### Inclusion Criteria

Studies were included if any of the following criteria were met: (1) reported outcomes of Perceval compared with other heart valve prostheses or procedures; (2) reported analysis of complications using the Perceval; (3) reported off-label experience; (4) reported learning curve analysis; (5) reported one or more case of SAVR with Perceval.

### Exclusion Criteria

Studies were excluded if any of the following criteria were met: (1) reported outcomes of exclusively other sutureless valves; (2) grouped outcomes of Perceval with other prostheses in the same cohort; (3) not published in the English language; (4) not published in a peer-reviewed journal; and (5) was a conference abstract.

### Data Collection

**Figure 1:**
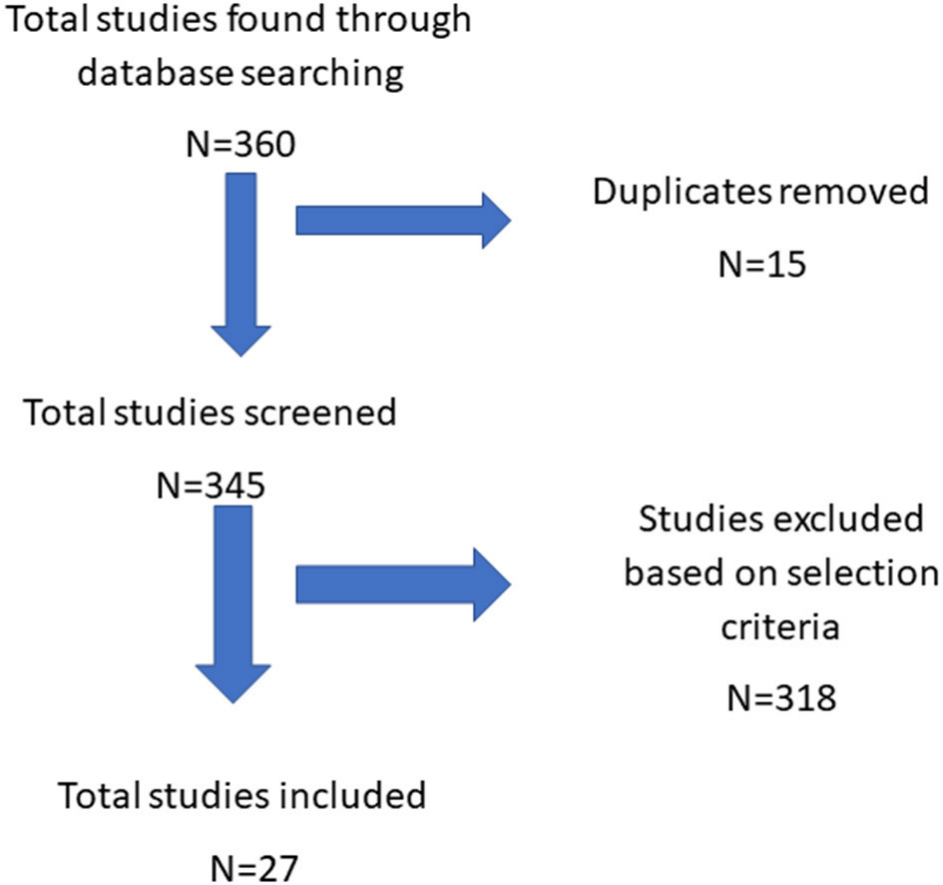
Flowchart study selection process.

The data collection was done on August 31, 2021. One author (AD) screened the articles and reviewed it three times. The final results were reviewed by another investigator (MPS). The primary reported outcomes of the study included (a) the surgical technique; (b) clinical trials investigating the Perceval valve; (c) the sutureless vs. TAVR; (d) the sutureless vs. other stented bioprostheses (e) Perceval in mini-SAVR; (f) Perceval and bicuspid aortic valves; (g) long-term outcomes of the Perceval valve (valve durability); (h) the incidence of thrombocytopenia after Perceval implantation; (i) the ideal candidate for the prosthesis implantation (Figure 1).

## The Most Appropriate Surgical Technique for Valve Implantation

The aortic incision is performed at the distal portion at the sinotubular junction to preserve a segment of ascending aorta above the prosthetic valve. The aortic valve should be excised at a position corresponding to the incision line of the native leaflets and the aortic annulus should be decalcified to prepare the implant site. A complete decalcification of the aortic annulus is not necessary. To ensure the correct positioning and orientation of the prosthesis, three guiding sutures are placed to act as reference for accurate alignment of the inflow portion of the prosthesis with the insertion plane of the native leaflets. For each valve sinus, one stitch is positioned immediately 2–3 mm below the lowest portion of the native leaflet resection line. On the prosthesis, each guiding suture is passed into a dedicated thread loop located at the midlevel of the inflow ring and aligned to the median part of the prosthetic sinuses. Once the prosthesis is connected to the three guiding suture, the release device is introduced into the aorta (11). In this context, the Perceval Livanova company recommend placing the guiding sutures 2–3 mm below the leaflet insertion line. Using this technique, Yanagawa et al. (12) found a PPI rate of 28%. Therefore, they modified the technique by placing the guiding sutures at the nadir of each cusp and not 2 to 3 mm below. After the modification, the PPI rate dropped to 0%. Nguyen et al. (13), recommend performing the transverse aortotomy ~3.5 cm above the level of the aortic annulus, and 0.5 cm above the sinotubular junction, to leave a free edge for closure of the aortotomy. In bicuspid aortic valves, the surgeon must recreate 3 nadirs that are positioned at ~120° to better manage the asymmetry of each cusp. To achieve this, the surgeon can use a commercial sizer with 120° markings to recreate a normal nadir. In addition, a dedicated balloon should be inserted into the prosthesis and inflated at a pressure of 4 atm for 30 sec.

## Clinical Trials

The “PERCEVAL TRIAL—Perceval S valve pilot study was performed in 30 high-risk patients who were scheduled for isolated SAVR due to severe aortic stenosis (14). This prospective analysis was undertaken at three European Centers from April 2007 to February 2008 and concentrated on perioperative and 1-year outcomes. Operative mortality was 3.3% and moderate paravalvular leak (PVL) was present in two patients. The PERCEVAL-AVR clinical trial evidenced the non-inferiority for the sutureless vs. stented for major adverse cerebral and cardiovascular events at 1 year, whereas aortic valve hemodynamics improved equally in both groups. Perceval significantly reduced surgical times (mean CPB: 71.0 ± 34.1 vs. 87.8 ± 33.9 mins; mean aortic cross-clamp times: 48.5 ± 24.7 vs. 65.2 ± 23.6; both *p*-values < 0.001), but resulted in a higher rate of permanent pacemaker implantation (PPI – 11.1 vs. 3.6% at 1 year). Incidences of PVL and central leak were similar.

The CAVALIER clinical trial (15) reported a mean cross-clamp time of 41.5 ± 20.3 mins and a mean CPB time of 39.0 ± 12.5 mins while the mean hospital length of stay was 12.0 ± 7.4 days. There were three reported cardiac valve-related deaths, and eight cases were cardiac related but not valve related. There were five early explanted valves 13.8 days post-implant due to PVL discovered at follow-up.

## Perceval vs. Tavr. When Enemies Become Allies

**Figure 2:**
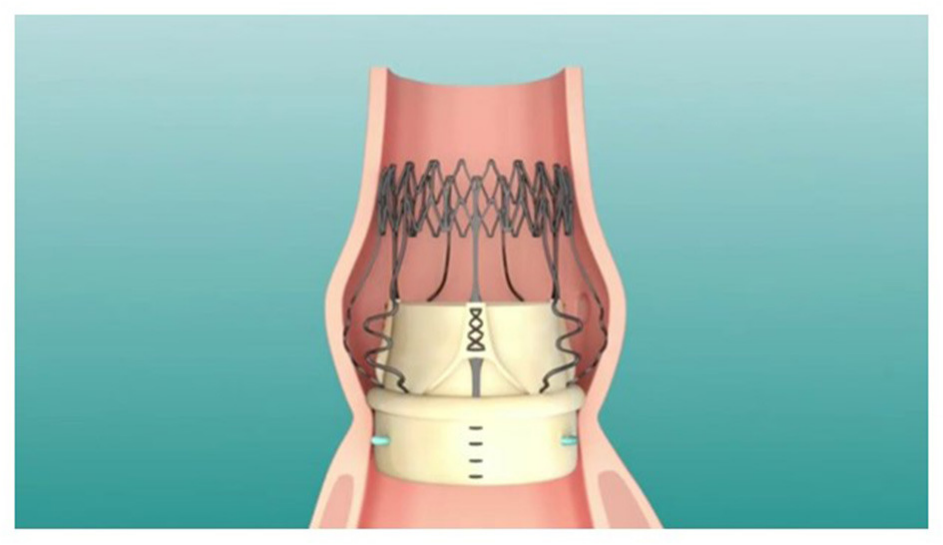
Sutureless aortic valve in the aortic annulus.

SVD has been reported in many case series and the treatment in these patients has successfully been delivered through valve-in-valve TAVR using both the Evolut Pro and the Corevalve (16) (Figure 2). With respect to Perceval vs. TAVR, the SURTAVI trial (6) showed that TAVR with the self-expanding CoreValve was non-inferior to SAVR for the primary endpoint at 2 years for the treatment of severe aortic stenosis in intermediate-risk patients (STS-PROM, 3–15%; median 4.5%). The Perceval valve benefits, may render ViV-TAVR second procedure easier and safer. This includes a self-expanding nitinol stage, a radio-opaque frame, and sinusoidal struts that “push” coronary ostia and sinuses away from prosthesis leaflets. In addition, eight retrospective clinical studies showed that 30-day mortality was higher in the TAVR group which may be explained with the higher preoperative risk in this population (16–24). The most used prosthesis in TAVR were the Corevalve, Sapien, Lotus and Portico. The CPB and aortic cross-clamp time for the Perceval ranged between 54 and 73.4/SD = 23.1–25 mins and 32–43.4/SD = 13.4–17, respectively. Mortality ranged from 0 to 4% for Perceval and 2.9–7% for TAVR. The incidence of PVL (Perceval 1.9–19.4 vs. TAVR 9–53.5%), PPI (Perceval 2–11.2 vs. TAVR 4.9–25.5%), stroke (Perceval 0 vs. TAVR 0–2.8%), and myocardial infarction (MI) (Perceval 0 vs. TAVR 0–3.5%), were all higher in the TAVR group (Table 1).

**Table 1:** Sutureless aortic valve replacement vs. transcatheter aortic valve replacement.

**Study author**	**Biancari et al**.		**Muneretto et al**.		**D'Onofrio et al**.		**Santarpino et al**.		**Miceli et al**.		**Muneretto et al**.		**Repossini et al**.		**Gerfer et al**.	
**Type of clinical study**	**Retrospective**		**Retrospective**		**Retrospective**		**Retrospective**		**Retrospective**		**Retrospective**		**Retrospective**		**Retrospective**	
**Valve types and nr. of patients**	**Perceval** ***N*** **= 144**	**TAVR** ***N*** **= 144**	**Perceval** ***N*** **= 53**	**TAVR** ***N*** **= 55**	**Perceval** ***N*** **= 31**	**TAVR** ***N*** **= 143**	**Perceval** ***N*** **= 443**	**TAVR** ***N*** **= 1,002**	**Perceval** ***N*** **= 37**	**TAVR** ***N*** **= 37**	**Perceval** ***N*** **= 288**	**TAVR = 367**	**Perceval = 158**	**TAVR = 158**	**Perceval = 59**	**TAVR = 59**
30-day Mortality (%)	1.4	6.9	0	1.8	0	7	4	2.9	0	3	5.8	9.8	1.9	5.8	5.1	1.7
Bleeding (%)	4.2	0	7.5	0	NR	NR	NR	NR	1	1	4.9	1.9	NR	NR	NR	NR
Paravalvular leak (%)	2.8	53.5	1.9	9	19.4	28.7	NR	NR	2	30	4	18	0.5	4.3	0	6.8
Stroke (%)	0	2.1	0	0	0	2.8	NR	NR	0	2	1.5	5.8	NR	NR	1.7	0
Myocardial Infarction (%)	0	0	0	1.8	0	3.5	NR	NR	NR	NR	NR	NR	NR	NR	NR	NR
Permanent pacemaker implantation (%)	11.2	15.4	2	25.5	3.2	4.9	5.8	11.6	2	0	9.8	14.7	5.4	11.9	10.2	8.5
Aortic cross-clamp time in minutes ± SD	42 ± 17	NA	32 ± 14	NA	NR	NA	43.4 ± 13.4	NA	NR	NA	32.8 ± 12.6	NA	NR	NR	49 ± 22	NA
Cardiopulmonary bypass time in minutes ± SD	71 ± 24	NA	54 ± 25	NA	NR	NA	73.4 ± 23.1	NA	NR	NA	50 ± 11.5	NA	NR	NR	83 ± 32	NA
TAVR types	NA	CoreValve Sapien Lotus Portico	NA	NR	NA	NR	NA	Sapien	NA	Sapien	NA	Corevalve, Sapien XT, Accurate TA	NA	NR	NA	Accurate NEO

*TAVR, transcatheter aortic valve replacement; SD, standard deviation; NR, not reported; NA, not applicable*.

## Perceval vs. Other Stented Bioprostheses. New Generation vs. Old Style

Compared to other stented bioprostheses (SB), the Perceval valve had similar outcomes. Four prospective and four retrospective clinical studies showed that 30-day mortality was higher in the Perceval group which may be explained with the higher preoperative risk in this population (24–31). Mortality ranged from 0 to 6.4% for Perceval and 0–5.9% for SB. The aortic cross-clamp time in minutes (Perceval 30.8–65.3/SD = 13.6–29.1 vs. SB 59–90/SD = 23–30.3) and CPB time in minutes (Perceval 47–88/SD = 11–34.9 vs. SB 87.8–120/SD = 20.4–37.9) were all significantly higher in the SB group (*p* < 0.05). The incidence of PVL (Perceval 1–19.4 vs. SB 0–1%), PM (Perceval 2–10.7 vs. SB 1.8–8.5%), stroke (Perceval 0–3.7 vs. SB 1.8–7.3%), MI (Perceval 0–7.8 vs. SB 0–4.3%), were comparable among the groups (Table 2). The most used stented valves were the CE Perimount, Magna Ease and Triflecta valves.

**Table 2:** Sutureless aortic valve replacement vs. other stented bioprostheses.

**Study author**	**Muneretto et al**.		**Gilmanov et al**.		**Pollari et al**.		**D'Onofrio et al**.		**Vaquero et al**.		**Fischlein et al**.		**Dalen et al**.		**Forcillo et al**.	
**Type of clinical study**	**Prospective**		**Retrospective**		**Prospective**		**Retrospective**		**Prospective**		**Prospective**		**Retrospective**		**Retrospective**	
**Valves and patients**	**Perceval** ***N*** **= 53**	**Stented** ***N*** **= 55**	**Perceval** ***N*** **= 133**	**Stented** ***N*** **= 133**	**Perceval** ***N*** **= 88**	**Stented** ***N*** **= 88**	**Perceval** ***N*** **= 31**	**Stented** ***N*** **= 112**	**Perceval** *N* **= 140**	**Stented** ***N*** **= 409**	**Perceval** ***N*** **= 447**	**Stented** ***N*** **= 449**	**Perceval** **= 171**	**Stented** **= 171**	**Perceval** **= 76**	**Stented = 319**
30-day Mortality (%)	0	0	0.8	1.5	2.4	3.7	0	1.8	6.4	5.9	1	1	1.8	2.3	5	6
Bleeding (%)	7.5	10.5	6.8	3.8	2.4	6.1	NR	NR	NR	NR	4.4	6.3	4.1	6,4	8	8
Paravalvular leak (%)	1.9	0	NR	NR	NR	NR	19.4	1	3.6	0.5	1	0	0	1.2	0	0
Stroke (%)	0	1.8	NR	NR	3.7	7.3	0	0	2.9	2.7	1.5	1.9	2.3	1.2	0	5
Myocardial infarction (%)	0	0	1.5	0	NR	NR	0	0.9	7.8	4.3	1	1.5	NR	NR	0	0
Permanent pacemaker implantation (%)	2	1.8	NR	NR	6.1	8.5	3.2	0.9	10.7	2	10.6	3.2	9.9	2.9	17	8
Aortic cross-clamp time in minutes/SD	30.8 ± 13.6	65.3 ± 27.7	56	90	47 ± 16	59 ± 23	NR	NR	65.3 ± 29.1	77.2 ± 30.3	48.5 ± 24.7	65.2 ± 23.6	40 ± 15	65 ± 15	46	68
Cardiopulmonary bypass time in minutes/SD	47 ± 18.5	89.4 ± 20.4	88	120	71 ± 11	92 ± 33	NR	NR	81.3 ± 34.9	95.7 ± 37.9	71.0 ± 34.1	87.8 ± 33.9	69 ± 20	87 ± 20	60	85
Type of stented valves	NA	Perimount, Edwards	NA	CE Edwards, Medtronic, CE standard	NA	NR	NA	NR	NA	Triflecta	NA	NR	NA	CE Perimount	NA	CE, Medtronic, Mitroflow, St. Jude epic, St. Jude Biocor

*NA, not applicable; SD, standard deviation; NR, not reported*.

## Perceval for Mics and Mini-Savr

One of the benefits of the Perceval bioprosthesis is its widespread usage in mini-SAVR. Perceval has been developed in order to combine the best of two worlds, as they could facilitate the implantation while maintaining the benefits of SAVR. Currently, the upper ministernotomy (MS) and the right anterior thoracotomy (RAT) are the most common approaches for (mini-SAVR). Bonacchi et al. (32) evidenced the benefits of the valve in both MS and RAT. In addition, the international prospective registry (33) comparing MS with RAT showed an aortic cross-clamp time of 43 vs. 55 mins (*p* < 0.01), cardiopulmonary bypass time of 67 vs. 89 mins (*p* < 0.01) and a prosthesis implantation time of 15.5 vs. 12 mins (*p* = 0.014), respectively. In this context, the Sutureless and Rapid Deployment International Registry (34), pointed out the efficacy of the Perceval bioprosthesis in redo operations showing a mean cardiopulmonary bypass time of 95 ± 34.3 mins, an aortic cross-clamp time 57.8 ± 23.2 mins with 0% in hospital mortality, a 3.6% incidence of new PPI and 2.5% incidence of PVL. Recent technological developments have led to endoscopic aortic valve replacement. Vola et al. (35) reported the endoscopic SAVR with Perceval. Exposure was provided by four ports in the second, third, and fifth intercostal spaces with fem-fem CPB. Perceval was implanted with an aortic cross-clamp and CPB time of 80 and 166 mins, respectively. At 5-month follow-up, echocardiography was satisfactory. Balkhy et al. (36) reported the first in human robotic SAVR with Perceval. The patient was a 76-year-old male who underwent a combined procedure of coronary artery bypass surgery and SAVR. Two 8-mm arm ports were placed in the 1st and 3rd intercostal space at the midclavivular line. Aortic cross clamp lasted 86 mins. The patient was discharged on postoperative day 2 and at 6-month follow-up the patient was in good health.

## Bicuspid Aortic Valves and Perceval

This topic remains controversial among surgeons. Many clinical studies, including the PERSIST-AVR clinical trial (37), excluded patients with a congenital bicuspid aortic valve. Some reports suggested that the sutureless valves may increase the risk of PVL and/or potential dislocation related to BAV aortic root asymmetry (38). Nguyen et al. (13) emphasized that the most crucial point during surgery is to recreate three natural nadirs points positioned at 120° with the aim of recreating a circular annulus. Four retrospective clinical studies (13, 34, 39, 40) with a small population ranging between 11 and 88 patients evidenced a low mortality rate (0–4%) and PVL incidence (0–2.3%). However, there was a high incidence of PPI (0–20%), and stroke (0–8%) (Table 3). The mean aortic cross clamp time in minutes (39–55/SD = 3.1–14) and CPB time in minutes (54.5–80/SD = 4.4–22) were higher compared to non BAV procedures. These outcomes mean that despite recent surgical technique developments, PPI remain a hurdle for BAV patients undergoing SAVR with sutureless bioprostheses.

**Table 3:** Clinical outcomes of bicuspid aortic valve stenosis treated with sutureless valve.

**Study author**	**Durdu et al**.	**Nguyen et al**.	**Szecel et al**.	**Miceli et al**.
	**(mean ± SD)**	**(mean ± SD)**	**(mean ± SD)**	**(mean ± SD)**
**Number of patients**	***N*** **= 13 patients**	***N*** **= 25 patients**	***N*** **= 11 patients**	***N*** **= 88 patients**
Type of clinical study	Retrospective	Retrospective	Retrospective	Retrospective
30-day mortality (%)	0	4	0	1.6
Bleeding (%)	7.6	1	NR	3.1
Paravalvular leak (%)	0	0	0	2.3
Stroke (%)	7.6	8	0	4.2
Myocardial infarction (%)	0	0	0	NR
Permanent pacemaker implantation (%)	7.6	20	0	5.7
Aortic cross-clamping time in minutes/SD	40.3 ± 3.1	45.9 ± 14.0	39 ± 13	55
Cardiopulmonary bypass time in minutes/SD	54.5 ± 4.4	56.1 ± 14.9	66 ± 22	80

*NR, not reported; SD, standard deviation*.

## Thrombocytopenia. Do We Really Need to Correct It?

Several causes of platelet dysfunction have been speculated: (1) the detoxification process with homocysteic acid and the storage aldehyde-free solution; (2) the naked alloy stent; and (3) mechanical stress and turbulence, especially in small valve sizes (41). At the end of the day, Vendramin and Bortolotti correctly pose the following questions: Do we really need to solve it and why should we still be worried (42)? In this context, Stegmeier et al. (43) showed that Perceval, when compared to other prostheses, is more prone to causing thrombocytopenia, however, no detrimental clinical effect of this phenomenon was found. The mean minimum platelets count was 47,000 μm and upon discharge the platelets level was 166,000 μm. Can medical therapy have an impact on thrombocytopenia? The result from the study showed a non-significant difference among patients on aspirin and dual antiplatelet medical therapy. In addition, there was no significant change in platelets and red blood cells transfusion. However, the reoperation for bleeding rate (20%) was higher than in the other two groups (Labcor TLPB-A = 4% and Hancock valve = 8%). Moreover, a sub-analysis of the PERSIST-AVR clinical trial evidenced that the Perceval group had a higher platelet reduction than the control group (46 vs. 32%) (44). The phenomenon was transient in both groups, with a slow recovery of the platelet count by hospital discharge. No differences were observed between groups regarding need of transfusions, blood loss, major bleeding and stroke events. While comparing the Intuity valve with its Perceval counterpart, Jiritano et al. (41) found that no risk factors that may have predisposed to platelet dysfunction were found in either group. More red blood cell transfusions were given to the Perceval group as compared with the Intuity group (10 vs. 7 units, *p* = 0.012) as well as platelets (4 vs. 0 units, *P* < 0.01). Platelet count at discharge for Perceval was 102.18 ± 29.34 μm. In addition, mean platelet volume was significantly larger in the Perceval group on postoperative days 1, 3, and 5 (*P* = 0.04, *P* = 0.001, *P* = 0.015), whereas platelet distribution width was significantly larger in the Perceval group on postoperative days 3 and 5 (*P* = 0.018, *P* = 0.026). Looking at the clinical studies outcomes the answer to Vendramin and Bortolotti is the following: “no, we do not need to correct the transient thrombocytopenia, but we should be cautious.”

## Hemodynamic Changes, Ventricular Mass Regression, and Porcelain Aorta

We found nine clinical studies but only eight were reporting data with standard deviations. Six of the studies were retrospective observational cohort studies and two were prospective non-randomized clinical trials (Table 4) (11, 21–23, 35, 36). The effective orifice area (EOA) ranged between 1.5 and 1.7 cm^2^/SD = 0.3–0.5 since discharge up to 2 years of follow-up. The mean transvalvular gradient ranged between 10.1 and 14 mmHg/ SD = 4.3–6.4 at discharge, 8.9 mmHg/ SD = 3.2–4.2 at 6 months, 8.7–9.9 mmHg/SD = 3.7–5 at 1 year and 8–9 mmHg/SD = 3.4–4.1 at 2 years follow-up. The peak transvalvular gradient was 19.4–27 mmHg/SD = 8.1–11 at discharge, 16.8–19.6 mmHg/SD = 6.7–7.6 at 6 months, 17.1–20.9 mmHg/SD 7.6–9.2 at 1 year, 16.6–18.3 mmHg/SD 5.6–7.2 mmHg at 2 years follow-up. With respect to the ventricular mass regression, Santarpino et al. (45) found that the mean ± SD left ventricular mass index decreased from 148.4 ± 48.4 g/m^2^ to 119.7 ± 38.5 g/m^2^ (P = 0.002) whereas interventricular septum and posterior wall thickness decreased from 13.9 ± 2.3 mm to 12.1 ± 2.8 mm (*P* = 0.02) and 12.1 ± 1.6 mm to 11.3 ± 1.3 mm (P = 0.04) at follow-up. In addition, there have been sporadic reports of the implantation of the Perceval in porcelain aortas. Santarpino et al. (46) reported a 72-year-old woman with severe AS, coronary artery disease, and porcelain aorta. The patient underwent CABG, removal of the ascending aorta, and implantation of a 23-mm Perceval and FlowWeave Bioseal 24-mm prosthesis (Jotec, Hechingen, Germany). Gatti et al. (47) reported the use of Perceval in four patients with porcelain aorta. All patients were discharged within postoperative day 20 and, at 1 to 6-month, were alive with improvements in symptoms.

**Table 4:** Hemodynamic outcomes.

**Endpoints**	**Santarpino et al.** ***N*** **= 658 (mean ± SD)**	**Rubino et al.** ***N*** **= 314** **(mean ± SD)**	**Mazine et al.** ***N*** **= 215** **(mean ± SD)**	**Folliguet et al.** ***N*** **= 208** **(mean ± SD)**	**Shrestha et al.** ***N*** **= 30** **(mean ± SD)**	**Shrestha et al.** ***N*** **= 243** **(mean ± SD)**	**Miceli et al.** ***N*** **= 37** **(Mean ± SD)**	**Repossini et al.** ***N*** **= 158**
Type of clinical study	Prospective	Retrospective	Retrospective	Retrospective	Prospective	Retrospective	Retrospective	Retrospective
EOA (cm^2^) at discharge	1.5 ± 0.4	NR	1.56 ± 0.37	1.4 ± 0.4	NR	1.5 ± 0.4	NR	NR
EOA (cm^2^) at 6 months	1.5 ± 0.3	NR	NR	1.5 ± 0.4	NR	1.5 ± 0.4	NR	NR
EOA (cm^2^) at 1 year	1.5 ± 0.4	NR	NR	1.5 ± 0.3	1.55 ± 0.35	1.6 ± 0.4	NR	NR
EOA (cm^2^) at 2 years	NR	NR	NR	NR	1.51 ± 0.26	1.7 ± 0.5	NR	NR
Mean gradient (mmHg) at discharge	10.3 ± 4.5	14 ± 6	13.3 ± 6.4	10.4 ± 4.3	NR	10.1 ± 4.7	11.4 ± 3.7	10.9 ± 5.4
Mean gradient (mmHg) at 6 months	8.9 ± 4.1	NR	NR	8.9 ± 3.2	NR	8.9 ± 4.2	NR	NR
Mean gradient (mmHg) at 1 year	9.2 ± 5	NR	NR	8.7 ± 3.7	9.9 ± 4.6	8.9 ± 4.6	NR	NR
Mean gradient (mmHg) at 2 years	NR	NR	NR	NR	8 ± 4.1	9 ± 3.4	NR	NR
Peak gradient (mmHg) at discharge	19.4 ± 8.1	27 ± 11	24.5 ± 10.8	21.3 ± 8.6	NR	20.3 ± 9.9	19.2 ± 6.9	18.7 ± 9.1
Peak gradient (mmHg) at 6 months	16.8 ± 7	NR	NR	19.6 ± 6.7	NR	18 ± 7.6	NR	NR
Peak gradient (mmHg) at 1 year	17.1 ± 8.7	NR	NR	18.8 ± 7.6	20.9 ± 9.2	17.5 ± 8.2	NR	NR
Peak gradient (mmHg) at 2 years	NR	NR	NR	NR	16.6 ± 7.2	18.3 ± 5.6	NR	NR

*EOA, effective orifice area; SD, standard deviation; NR, not reported*.

## Long-Term Outcomes Of The Perceval Valve

The Perceval aortic valve has proven to be a reliable bioprosthesis with excellent early and midterm outcomes. However, the long-term outcomes of the valve have not been studied and results are coming from some clinical studies. Our literature research found one retrospective study and one clinical trial with a 5-year follow-up period (Table 5). Shrestha et al. (48) reported the outcomes of 720 patients evidencing a 1.4% of cardiac deaths, 1,5% of valve explants, 1% of major paravalvular leak, 1.4% of A-V block III and 0.8% of stroke. The 5-year outcomes of a prospective clinical trial (14) with only 30 patients evidenced a cardiac mortality of 3.3%, an A-V block type III of 3.3% but no stroke, paravalvular leak, valve thrombosis or structural valve deterioration was noticed. The echocardiographic outcomes at 3, 4, and 5-year follow-up evidenced an EOA of 1.64–1.68 (SD 0.4–0.42), 1.68 (SD 0.43), 1.69–1.8 (SD 0.3–0.42), respectively. In addition, the mean transvalvular gradient across the valve at 3, 4, and 5 years was 7.7–8.3 mmHg (SD 2.5–2.8), 7.6–7.8 mmHg (SD 3.6–3.8), 8.8–9.3 mmHg (SD 4.6–5.5), respectively. These results once more confirm the usefulness of the Perceval valve (Table 6).

**Table 5:** Long-term outcomes of the Perceval bioprosthesis.

**Late events> 30 days.** **studies**	**Shrestha et al.** ***N*** **= 729 patients**	**Meuris et al.** ***N*** **= 30 patients**
Type of study	Retrospective	Prospective clinical trial
Follow-up duration	5 years	5 years
Deaths (%)	7	28.7
Cardiac deaths (%)	1.4	3.3
Valve explants (%)	1.5	0
Major paravalvular leak (%)	1	0
Endocarditis (%)	1.6	6.6
Structural valve deterioration (%)	0	0
Valve thrombosis (%)	0	0
AV block III (%)	1.4	3.3
Stroke	0.8	0

**Table 6:** Long-term echocardiographic outcomes (5-year follow-up) of the Perceval bioprosthesis.

**Study**	**Shrestha et al.** ***N*** **= 729 patients** **(mean ± SD)**	**Meuris et al.** ***N*** **= 30** **(mean ± SD)**
LVEF at 3 years (%)	67 ± 9	NR
LVEF at 4 years (%)	66.1 ± 9.1	NR
LVEF at 5 years (%)	65.8 ± 7.7	NR
Mean transvalvular gradient at 3 years mmHg	7.7 ± 2.8	8.3 ± 2.5
Mean transvalvular gradient at 4 years mmHg	7.8 ± 3.8	7.6 ± 3.6
Mean transvalvular gradient at 5 years mmHg	8.8 ± 4.6	9.3 ± 5.5
Peak transvalvular gradient at 3 years mmHg	16 ± 5.2	16.6 ± 6.2
Peak transvalvular gradient at 4 years mmHg	17.8 ± 8.1	17.5 ± 7.8
Peak transvalvular gradients at 5 years mmHg	21.1 ± 9.7	21.4 ± 11.5
EOA at 3 years (cm^2^)	1.64 ± 0.42	1.68 ± 0.4
EOA at 4 years (cm^2^)	1.68 ± 0.43	1.68 ± 0.43
EOA at 5 years (cm^2^)	1.8 ± 0.3	1.69 ± 0.42

## The Ideal Candidate for Sutureless Aortic Valve Replacement

Many studies have evidenced the benefits of Perceval aortic bioprosthesis, especially in the following three situations:

(a) High-risk patients undergoing a combined surgical procedure
(b) Hostile aortic root
(c) A small aortic annulus.

In the first situation, the use of sutureless and rapid-deployment valves allows economy of precious CPB time by alleviating the need to place and tie sutures around the aortic annulus, while still allowing native valve excision and annular decalcification. In a systematic review and meta-analysis that included 12 observational studies, Phan et al. (49) demonstrated that the pooled durations of cardiopulmonary bypass and aortic cross-clamp for isolated SAVR were 57 and 33 min, respectively. These values are nearly half of those reported in the Society of Thoracic Surgeons National Database^1^ for conventional SAVR.

In hostile aortic roots and redo operations, Perceval may become the bioprosthesis of choice. In addition to the time-saving procedure and to the non-necessity of complete annular decalcification, it allows valve replacement after graft infection. In the last scenario, the benefits include less foreign material used (pledgets/sutures), less manipulation of friable tissues, and radial force of Perceval solidifies root repair. During reoperations and extensive decalcification of the annulus, clefts in the mitral valve/left atrium can form. In this situation, the Perceval valve can be easily compressed and removed (without the necessity of removing all the sutures as in the stented valves), the cleft repaired, and the valve redeployed again (50). However, neither the CAVALIER nor the PERSISTENT-AVR clinical trials mentioned the hostile aortic root.

Finally, in case of a small aortic annulus, an aortic root enlargement should be performed to implant an adequately sized bioprosthesis. However, this is not always feasible as newly minted surgeons do not have sufficient technical experience to perform these procedures. In this scenario, the sutureless prosthesis have shown good outcomes when implanted with low post-procedural transvalvular gradients (45). In addition, Perceval is a proven option for high-risk patients and for those at risk of prosthesis-patient mismatch (51).

Contraindications for the prosthesis implantation are (a) subjects with aortic root enlargement, where the ratio between observed and expected diameters (calculated as a function of age and patient body surface area) is ≥1.3; (b) subjects with known hypersensitivity to nickel alloys, (c) subjects with aneurysmal dilation or dissection of the ascending aortic wall needing surgical correction.

## Potential Pitfalls of Perceval

Limitations and drawbacks of the Perceval bioprosthesis are the following;

(a) PVL.
(b) Acquired conduction disorders and PPI.
(c) SVD and need for reintervention.

PVL has shown an increased incidence in the TAVR and the sutureless bioprostheses with the latter being the highest (52). Surgeons came to understand that the Achilles heel of these bioprostheses is the non-coronary sinus which is slightly lower compared to the left and right sinuses. During the deployment phase, the valve must be positioned in a lower angle of 15–30° at the level of the non-coronary sinus, on the side of the surgeon. When the valve is accurately positioned, and no gap exists on visual inspection than it should be deployed. This technique avoids the incidence of PVL. However, it has been shown that these results are related to a learning curve and experienced surgeons tend to have a lower incidence of PVL (53).

The PPI trend has shown a slow but steadily decrease since the introduction by Yanagawa et al. (12) of their modification of the implantation height. They found that a higher implantation of the valve (2–3 mm) decreases the incidence of conduction abnormalities requiring a pacemaker. This is in contrast with the first prescription given from the company to implant the valve below the annular plane. SVD happens continuously and Perceval is not exempt from it.

## Conclusions

The Perceval bioprosthesis has proved to be a reliable prosthesis for conventional SAVR and mini-SAVR due to its implantation speed, the reduced CPB time, the reduced aortic cross-clamp time and the shorter intensive care unit and hospital length of stay. In addition, its adoption in hostile roots, and the usage in reinterventions coupled with the low profile render it a formidable tool in the surgical armamentarium.

## Author Contributions

All authors listed have made a substantial, direct, and intellectual contribution to the work and approved it for publication.

## Conflict of Interest

BR has received financial support from Medtronic, LivaNova, and AtriCure. The remaining authors declare that the research was conducted in the absence of any commercial or financial relationships that could be construed as a potential conflict of interest.

## Publisher's Note

All claims expressed in this article are solely those of the authors and do not necessarily represent those of their affiliated organizations, or those of the publisher, the editors and the reviewers. Any product that may be evaluated in this article, or claim that may be made by its manufacturer, is not guaranteed or endorsed by the publisher.

## Footnotes

^1^ https://publicreporting.sts.org/

## Abbreviations

TAVR: transcatheter aortic valve replacement
CPB: cardiopulmonary bypass
PPI: permanent pacemaker implantation
BAV: bicuspid aortic valve
MI: myocardial infarction
PVL: paravalvular leak
SAVR: surgical aortic valve replacement
MS: mini-sternotomy
RAT: right anterior thoracotomy.
